# Strategic Modification of Scallop Mantle Protein Isolates via Alkaline Extraction: Resource Utilization of Biological Waste

**DOI:** 10.3390/foods15183352

**Published:** 2026-09-21

**Authors:** Yao Xie, Yifei Zheng, Wenhong Cao, Mingtang Tan

**Affiliations:** 1 College of Food Science and Technology, Guangdong Ocean University, Zhanjiang 524088, China; xie1099184959@163.com (Y.X.); zyf431@stu.gdou.edu.cn (Y.Z.); cwenhong@gdou.edu.cn (W.C.); 2 Shenzhen Institute of Guangdong Ocean University, Shenzhen 518108, China; 3 National Research and Development Branch Center for Shellfish Processing (Zhanjiang), Guangdong Provincial Key Laboratory of Aquatic Products Processing and Safety, Guangdong Provincial Engineering Technology Research Center of Seafood, Zhanjiang 524088, China

**Keywords:** scallop byproducts, alkaline extraction, physicochemical properties, functional characteristics, seafood byproducts

## Abstract

Scallop mantle, an abundant yet underutilized processing byproduct, holds vast potential for high-value valorization, yet its restricted functional versatility hinders food-grade application. This study systematically analyzed the molecular mechanisms governing the characteristic evolution (physicochemical, structural, and functional) of scallop mantle protein isolates (SMPIs) subjected to alkaline extraction (pH 8–12). Results indicated that extraction pH from 8 to 12 dramatically boosted the yield of SMPIs from 5.01% to 96.29% and increased purity (reaching 96.01% at pH 10.0 before plateauing), alongside increased particle size and surface hydrophobicity, but caused a dark yellow appearance at pH 12. During this process, proteins gradually degraded into small peptide fragments, with α-helix progressively transforming into β-sheet structures. The exposed hydrophobic residues and altered surface charges substantially modulated aggregation, generating highly uniform and dense microstructures at elevated pH. Notably, SMPIs exhibited optimal water absorption capacity, emulsifying stability index (ESI), and foaming stability at extraction pH 10. SMPIs demonstrated superior performance in thermal stability and foaming ability at extraction pH 11. At extraction pH 8, the stable conformation of SMPIs conferred the highest solubility and superior oil absorption capacity. Our research findings will facilitate the utilization of scallop mantle as a customizable resource for specific functionalities under the waste-to-wealth paradigm.

## 1. Introduction

The growing global demand for protein is fueling the exploration and commercialization of new protein-rich foods. Presently, plants (e.g., beans, nuts) or terrestrial animals (e.g., eggs, milk) are the primary sources of protein [1]. Aquatic proteins are increasingly recognized by consumers globally for their well-balanced amino acid composition, superior digestibility, distinct flavor profile, and abundant micronutrient content [2]. According to information from the China Fisheries Statistical Yearbook, scallop aquaculture in China achieved production of 1.95 million tons in 2024, utilizing an area of 350,000 hectares [3]. The adductor muscle is the primary edible part of the scallop, commonly processed into dried scallop [4]. Unfortunately, the low-edible mantle accounts for about 10% of total scallop wet weight (excluding the shell), which is discarded and wasted indiscriminately as a low-value byproduct, with only a small portion being processed and reused [5]. Previous studies have demonstrated that the scallop mantle is rich in protein, polysaccharides, vitamin E, unsaturated fatty acids, calcium, zinc, and selenium, of which taurine content is 36.3–36.7 mg/g and collagen content accounts for 7.7–12.6% of dry tissue [6]. Therefore, the preparation of scallop mantle proteins with highly efficient functional characteristics offers a promising vision for aquatic waste management.

Scallop mantle protein is gaining increasing attention due to its high nutritional value and excellent functional properties. Zhi et al. [7] isolated antioxidant peptides from the scallop (*Argopecten irradians*) mantle using neutral proteases and microbial fermentation. Exosomes derived from scallop mantle enhanced osteogenesis and alleviated osteoporosis in mice with ovariectomy-induced osteoporotic conditions [8]. To enhance the structural and functional properties of proteins, various modification techniques have been explored. For instance, combination of ultra-high pressure and salt addition improved the in vitro digestibility of scallop mantle myofibrillar proteins [6]. Additionally, appropriate high hydrostatic pressure treatment could induce unfolding of scallop mantle proteins and enhanced dispersion behavior, thereby improving the protein emulsifying properties [5]. Ultrasound could modulate the water absorption, oil absorption, foaming, and emulsifying properties of scallop mantle proteins by regulating protein structural stability and amphiphilicity [9]. However, these methods typically require complex equipment and yield low recovery rates.

The alkaline solubilization–acid precipitation process is a cost-effective technique that offers high recovery rates and improved functional properties [10]. The increased electrostatic repulsion between protein molecules in alkaline or acidic environments causes conformational unfolding and protein solubilization. However, the protein molecules rapidly reaggregate and precipitate when the pH is adjusted from alkaline or acidic conditions to the protein’s isoelectric point [11]. Previous studies have elucidated that the alkaline solubilization–acid precipitation method not only enhanced the utilization efficiency of tilapia protein but also improved the stability of tilapia protein isolate emulsion [12]. Furthermore, this processing step notably elevates the functional attributes of proteins from both plant and animal sources, including digestibility [13], gelling capacity [14], and emulsifying properties [15]. Research by Liu et al. [1] revealed that extraction pH altered the secondary structure and tryptophan microenvironment of oyster protein isolate (OPI), with OPI extracted at pH 10 exhibiting the highest heat resistance, foaming capacity, and gel strength. As a byproduct of scallop processing, the main protein types found in scallop mantle differ significantly from those in oysters. Even when the same alkaline extraction method is used, this may result in differing functional characteristics.

Although numerous reports have highlighted the critical importance of alkaline pH for protein extraction efficiency and functional properties, the effects of pH selection on the conformation and functional characteristics of scallop mantle protein isolates remain limited due to species-specific variations. Therefore, the objective of this study was to investigate the effects of alkaline extraction (from pH 8 to pH 12) on the physicochemical, structural, and functional properties of scallop mantle protein isolates (SMPIs). This research will enhance the recovery efficiency of scallop mantle protein and drive the transformation of waste into high-quality products.

## 2. Materials and Methods

### 2.1. Materials

The scallop (*Argopecten irradians*) was harvested from the sea area of Zhanjiang, China (21.8° N, 110.17° E). The scallop mantle was provided by a local scallop processing plant. To ensure sample consistency and minimize individual biological variations, a single batch of fresh scallop mantle was utilized. The samples were sub-packaged with 500 g in each resealable bag and transported to the laboratory in an ice container. All chemicals were of analytical grade.

### 2.2. Extraction of Scallop Mantle Protein Isolates

The scallop mantle protein isolates (SMPIs) were extracted according to the method described by Liu et al. [1]. The chopped scallop mantle (500 g/pH) was dispersed in ultrapure water (1:3, *w*/*v*) to form a suspension. The suspension was homogenized for 2 min at 10,000 rpm using a homogenizer and then adjusted to the desired pH values (8, 9, 10, 11, and 12) with 2 mol/L NaOH. After pH adjustment, the solution was stirred on an ice bath for 2 h, followed by centrifugation (13,800× *g*, 15 min) at 4 °C. Each protein solution was adjusted to 5.5 using 2 mol/L HCl and then stood at 4 °C for 1 h. The mixture was then centrifuged again at 4 °C (13,800× *g*, 15 min). The precipitate obtained was redispersed in ultrapure water (1:2, *w*/*v*) and adjusted to neutral (pH 7.0) using 2 mol/L NaOH. Finally, the SMPIs were freeze-dried and stored at −20 °C for subsequent experiments. The treated SMPIs were designated as pH 8, pH 9, pH 10, pH 11, and pH 12 based on their respective pHs. While freeze-drying-induced dehydration stresses might exert minor impacts on surface protein aggregation, all samples underwent standardized processing, ensuring that the observed functional variations reliably reflect the governing influence of extraction pH. Notably, for each pH value, three separate samples (each weighing 500 g) were selected and subjected to a complete extraction process.

### 2.3. Protein Yield and Purity

The determination of SMPI yield and purity followed the method described by Liu et al. [1]. The Biuret method was used to estimate the protein content of SMPIs using bovine serum albumin as the standard. The SMPIs’ yield and purity were estimated using Equations (1) and (2).

(1) $$Yield\;\left(\%\right)=\frac{isolate\;protein\;content\;\left(\%\right)\times isolate\;weight\;\left(g\right)}{mantle\;protein\;content\;\left(\%\right)\times mantle\;weight\;\left(g\right)}\times 100$$

(2) $$Purity\;\left(\%\right)=isolate\;protein\;content\;\left(\%\right)$$

### 2.4. Determining the Physicochemical Properties of SMPIs

#### 2.4.1. Protein Molecular Weight Distribution

The molecular weight distribution of SMPIs was analyzed using sodium dodecyl sulfate polyacrylamide gel electrophoresis (SDS-PAGE), following the method previously described [16]. Briefly, the 160 μL SMPIs (2 mg/mL of protein content) were mixed with 40 μL of protein loading buffer (5×). The mixture was heated in boiling water for 5 min and then centrifuged at 9000× *g* for 3 min. Then, 10 μL of the supernatant was loaded into electrophoresis gel lanes consisting of a 5% concentrating gel and a 12% separating gel, along with the marker. After completion of electrophoresis, the protein bands were stained with Coomassie Brilliant Blue Rapid Staining Solution, followed by decolorizing in a Coomassie Brilliant Blue Rapid Decolorizing Solution.

#### 2.4.2. Color and Visible Appearance

The color of SMPI powder was assessed with an NS800 chromaticity analyzer (3NH Technology Co., Ltd., Shenzhen, China), which was calibrated with standard black and white calibration plates [1]. The visual appearance of the sample was analyzed using images captured using an iPhone 16 Pro Max (Apple Corporation, Cupertino, CA, USA).

#### 2.4.3. Microstructure

The appropriate amount of freeze-dried SMPI powder was evenly adhered to the conductive adhesive, and a layer of gold nanoparticles was sprayed onto it. The specimens were then observed and photographed using a scanning electron microscope (FEI XL-30, Philips Corporation, Wetzlar, Germany) at a voltage of 6 kV. The magnification levels were 200×, 800×, and 2000× [17].

#### 2.4.4. Differential Scanning Calorimetry (DSC)

The thermal properties of SMPIs were measured through differential scanning calorimetry (DSC-8500, PerkinElmer, Waltham, MA, USA), as previously described by Tan et al. [18]. First, 5 mg of lyophilized protein sample was placed in an aluminum crucible and sealed, with an empty sealed pan used as a thermal reference. DSC measurement conditions were as follows: the sample was first held at 20 °C for 1 min and then heated from 20 °C to 105 °C at a rate of 5 °C/min under a nitrogen flow of 10 mL/min. The maximum denaturation temperature (T_max_) was determined from the obtained thermal profile data using the analysis software integrated with the instrument. Each sample was measured in triplicate.

#### 2.4.5. Particle Size Distribution, Polydispersity Index, and Zeta Potential

The particle size distribution, polydispersity index (PDI), and zeta potential of the SMPIs were determined using the Zetasizer ZEN3700 (Malvern Instruments, Worcestershire, UK) [11]. The SMPIs were prepared and mixed with ultrapure water (1.0 mg/mL of protein content, pH 7). The dispersions were centrifuged before measurement at 2000× *g* for 3 min. All samples were tested at 25 °C, with three replicates conducted.

### 2.5. Determining the Structural Properties of SMPIs

#### 2.5.1. Surface Hydrophobicity

According to previous studies [19] with modifications, the bromophenol blue (BPB) binding method was used to detect the surface hydrophobicity of SMPIs. First, 1.0 mL of protein solution (1.0 mg/mL of protein content) was thoroughly mixed with 200 μL of BPB solution (1.0 mg/mL) for 10 min. Subsequently, the mixture was centrifuged at 5000× *g* for 20 min. Then, 1.0 mL of supernatant was diluted with 9.0 mL distilled water, and absorbance (A_sample_) was measured at 595 nm. The solution containing 1.0 mL of distilled water and 200 μL of BPB solution (1.0 mg/mL) served as the blank sample, denoted as A_blank_. Surface hydrophobicity was calculated as

(3) $$\mathrm{Surface}\;\mathrm{hydrophobicity}\;\left(\mu g\right)=200\times \frac{A_{\mathrm{blank}}-A_{\mathrm{sample}}}{A_{\mathrm{blank}}}$$

#### 2.5.2. Free and Total Sulfhydryl Contents

The determination of free and total sulfhydryl contents in SMPIs followed the procedure described by Liu et al. [16] with some modifications. The free sulfhydryl groups were determined as follows: 1.5 mL of SMPI sample solution (5 mg/mL of protein content) mixture with 10 mL of Tris-Gly buffer (0.086 mol/L Tris, 0.09 mol/L Glycine, 4 mmol/L EDTA, pH 8.0) added to 50 μL of Ellman’s reagent (Tris-Gly buffer, pH 8.0, 4 mg/mL DTNB) to react for 1 h at 25 °C in the dark. Following centrifugation (8000× *g*, 10 min, 4 °C), supernatant absorbance was measured at 412 nm using the Tris-Gly buffer as a blank. The free sulfhydryl content was determined with a molar extinction coefficient of 13,600 M^−1^·cm^−1^. Unlike measurements of free sulfhydryl content, the buffer used to measure total sulfhydryl content contained 8 M urea.

(4) $$SH\left(\frac{\mu mol}{g}\right)=\frac{73.53\times A_{412}\times D}{C}$$

where A_412_ represents the absorbance value at 412 nm, D is the dilution factor, and C denotes the protein concentration (mg/mL).

#### 2.5.3. FTIR Spectra

FTIR spectra were recorded using an FTIR spectrometer (TENSOR 27, Bruker Corporation, Billerica, MA, USA) following the potassium bromide compression method [20]. The resolution was established at 32 scans with a bandwidth of 4 cm^−1^ covering a wave number range from 400 to 4000 cm^−1^. Each spectrum underwent calibration, and the Amide I region (1600–1700 cm^−1^) was analyzed using PeakFit software (v4.12, Systat Software Inc., San Jose, CA, USA) to assess the protein secondary structure, including the proportions of α-helix, β-sheet, β-turn, and random coil conformations.

#### 2.5.4. Intrinsic Fluorescence Spectroscopy

Fluorescence spectroscopy (RF-5301PC, Shimadzu, Kyoto, Japan) was applied to analyze intrinsic fluorescence spectra of SMPIs (0.1 mg/mL of protein content) according to the method described by Zhou et al. [21]. Among them, the excitation wavelength was 283 nm and the emission wavelength was 280–450 nm within the slit width of 5 nm.

### 2.6. Determining the Functional Properties of SMPIs

#### 2.6.1. Solubility

The solubility of SMPIs was determined following the method outlined by Liu et al. [1]. SMPIs were dissolved in ultrapure water (5 mg/mL of protein content, pH 7) and centrifuged (9000× *g*, 20 min, 4 °C). The protein content in the supernatant was determined using the Biuret method. The solubility was expressed as the percentage of protein content in the supernatant according to the equation

(5) $$\mathrm{Solubility}\;(\%)=\frac{\mathrm{supernatant}\;\mathrm{protein}\;\mathrm{content}\;\left(\mathrm{mg}/\mathrm{mL}\right)}{\mathrm{solution}\;\mathrm{total}\;\mathrm{protein}\;\mathrm{content}\;\left(\mathrm{mg}/\mathrm{mL}\right)}\times 100$$

#### 2.6.2. Emulsifying Properties

The emulsifying activity index (EAI) and emulsifying stability index (ESI) of SMPIs were determined following the method described by Yu et al. [22]. Briefly, 3 mL of soybean oil was added to 9 mL of protein solutions (15 mg/mL of powder mass, pH 7). The mixture was homogenized at 24,000 rpm for 30 s at room temperature. Subsequently, 50 μL of emulsion was collected from the bottom of the mixture at 0 min and 30 min, respectively, and mixed with 5 mL of 0.1% (*w*/*v*) SDS solution. With SDS solution serving as the blank control, absorbance values were measured at 500 nm using a spectrophotometer. The calculation formula of EAI and ESI is shown as follows:

(6) $$\mathrm{EAI}\;(m^{2}/g)=\frac{2\times 2.303\times A_{0}\times D}{C\times \varphi \times (1-\theta )\times 10^{4}}$$

(7) $$\mathrm{ESI}\;(\min)=\frac{A_{0}}{A_{0}-A_{30}}\times 30$$

where D represents the dilution factor (D = 100), C is the protein concentration (g/mL), φ is the path length (φ = 1 cm), θ represents the volume fraction of the oil phase (0.25), and A_0_ and A_30_ refer to the absorbance of the sample at 0 min and 30 min, respectively.

#### 2.6.3. Water Absorption Capacity (WAC) and Oil Absorption Capacity (OAC)

The WAC and OAC of SMPIs were evaluated using the methodology outlined by Nouska et al. [23] with slight modifications. For this analysis, 0.1 g of SMPI was accurately weighed into a 10 mL centrifuge tube and added to 5 mL of water (or soybean oil). The mixtures were vortexed for 1 min, rested for 30 min, and centrifuged at 5000× *g* for 20 min at 4 °C. The supernatant was discarded, and the WAC and OAC were calculated according to the following formula:

(8) $$\mathrm{WAC}\;\mathrm{or}\;\mathrm{OAC}\;(g/g)=\frac{W_{1}-W_{0}}{W_{0}}$$

where W_1_ is the weight of the centrifuged sample (g) and W_0_ is the initial weight of the sample (g).

#### 2.6.4. Foaming Properties

The foaming capacity (FC) and foaming stability (FS) of SMPIs were evaluated according to a previous study [24]. A 20 mL suspension of SMPIs (10 mg/mL of powder mass, pH 7) was added to a 50 mL volumetric cylinder and homogenized for 2 min at 10,000 rpm. The total volume of foam at t = 0 min and 10 min after homogenization was calculated as V_0_ and V_10_. The FC and FS were calculated as follows:

(9) $$\mathrm{FC}\;(\%)=\frac{V_{0}}{20}\times 100$$

(10) $$\mathrm{FS}\;(\%)=\frac{V_{10}}{V_{0}}\times 100$$

### 2.7. Statistical Analysis

Each experiment was conducted in triplicate to verify consistency (each replicate represents an independently prepared batch). Results are expressed as mean ± standard deviation (SD). Statistical analyses were conducted using IBM SPSS Statistics 26 software. A significance threshold of *p* < 0.05 was established as analyzed through one-way analysis of variance (ANOVA) followed by Duncan’s multiple range test.

## 3. Results and Discussion

### 3.1. Yield and Purity Analysis

**Figure 1:**
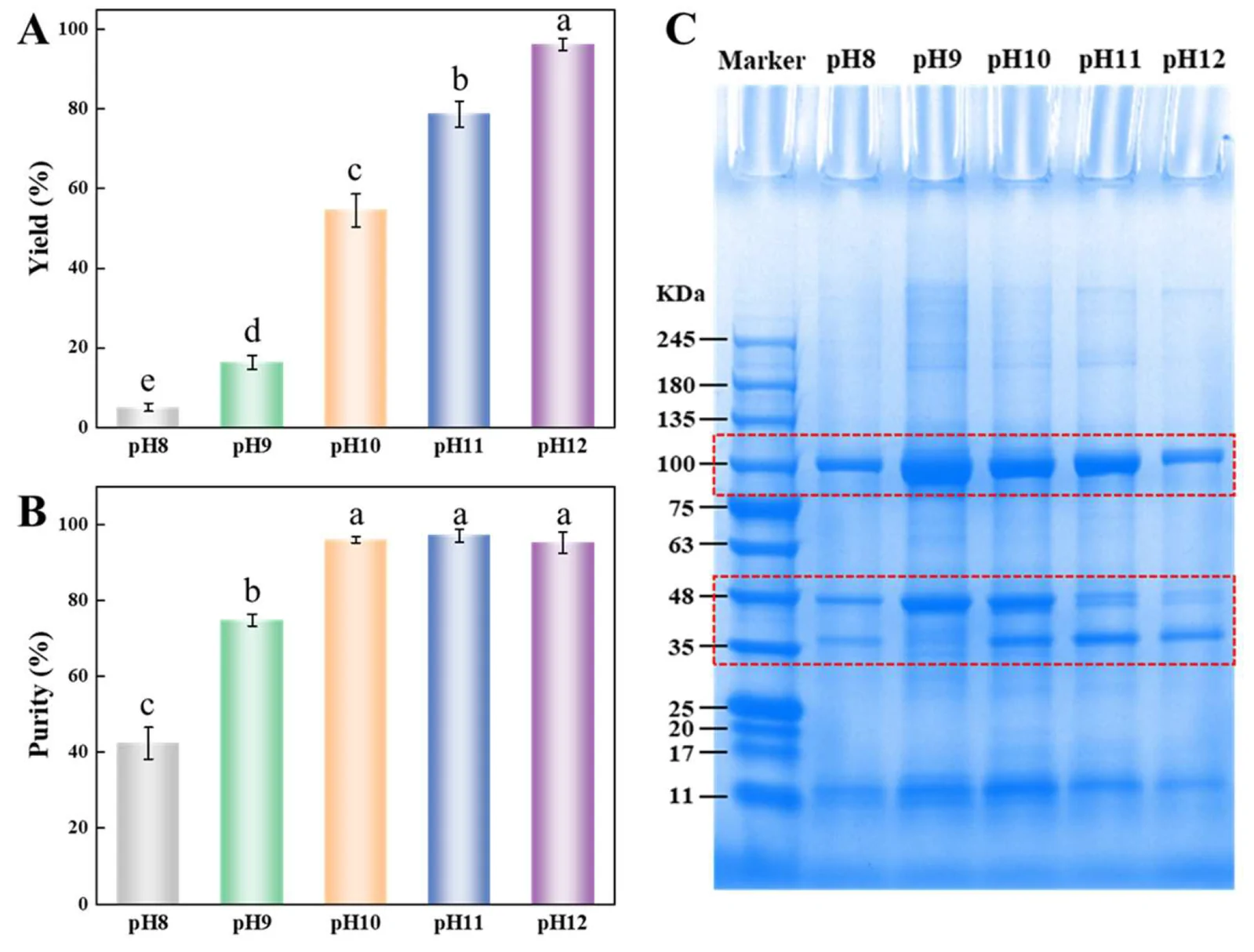
Yield (**A**), purity (**B**), and SDS-PAGE (**C**) of scallop mantle protein isolates with different extraction pH. Different letters indicate significant differences (*p* < 0.05).

The yield and purity of SMPIs were significantly affected by the extraction pH (*p* < 0.05), as shown in Figure 1A,B. As pH increased, SMPIs’ yield significantly increased (*p* < 0.05), achieving the highest yield at pH 12 (96.29%). An increase in extraction pH likely disrupted the hydrogen bonds between the carboxyl and sulfate groups in the cell membrane, thereby enhancing protein yield [25]. Furthermore, the high protein yield was attributed to the pH enhancing protein solubility due to the ionization of the carboxylic groups and de-protonation of the amine group. Notably, the alkaline conditions in this study exerted a far greater impact on SMPI yield than those observed in amaranth seeds (12.78–52.08%), lanternfish (49.43% at pH 12), and oysters (20.70–65.87%) [1,26,27]. Therefore, alkaline conditions were favorable for SMPI extraction, particularly at pH ≥ 10. Moreover, the purity of SMPIs increased with rising extraction pH, although no significant differences were observed at pH above 10 (*p* > 0.05). It had been reported that protein purity may decrease at higher extraction pH because of co-precipitation of non-protein components [25]. It may likewise contribute to the presence of more residual salt when subjected to extraction at a higher pH. Hence, we observed that purity had not shown an increasing trend even as the yield gradually increased with rising pH once the extraction pH exceeded 10. The present findings were consistent with our previous study on oyster protein isolates [1].

### 3.2. SDS-PAGE Analysis

The effect of pH on the composition of scallop mantle protein isolates (SMPIs) was analyzed using SDS-PAGE. As shown in Figure 1C, the major proteins in SMPIs were paramyosin (100 kDa), actin (48 kDa), tropomyosin (35 kDa), and myosin light chain (11 kDa) of myofibrillar proteins. This was different from the composition of scallop mantle protein (pH 7) reported by Ding et al. [5]. We noted that the paramyosin band in this study was particularly prominent, suggesting that alkaline extraction may lead to decreased paramyosin band intensity. Furthermore, the actin and myosin light chain band intensity exhibited reduced intensity and narrowing as the extraction pH increased from 9 to 12. This indicated that extraction pH substantially affected the molecular weight distribution of SMPIs and caused degradation. This may be due to alkali-induced protein degradation resulting in a corresponding increase in small molecular subunits (tropomyosin). Similarly, Xu et al. [28] reported that the macromolecular subunits of protein isolates gradually decreased with increasing alkaline pH, while the small molecular subunits of 28 and 30 kDa gradually increased in silkworm pupa protein isolates.

### 3.3. Color and Visible Appearance Analysis

**Figure 2:**
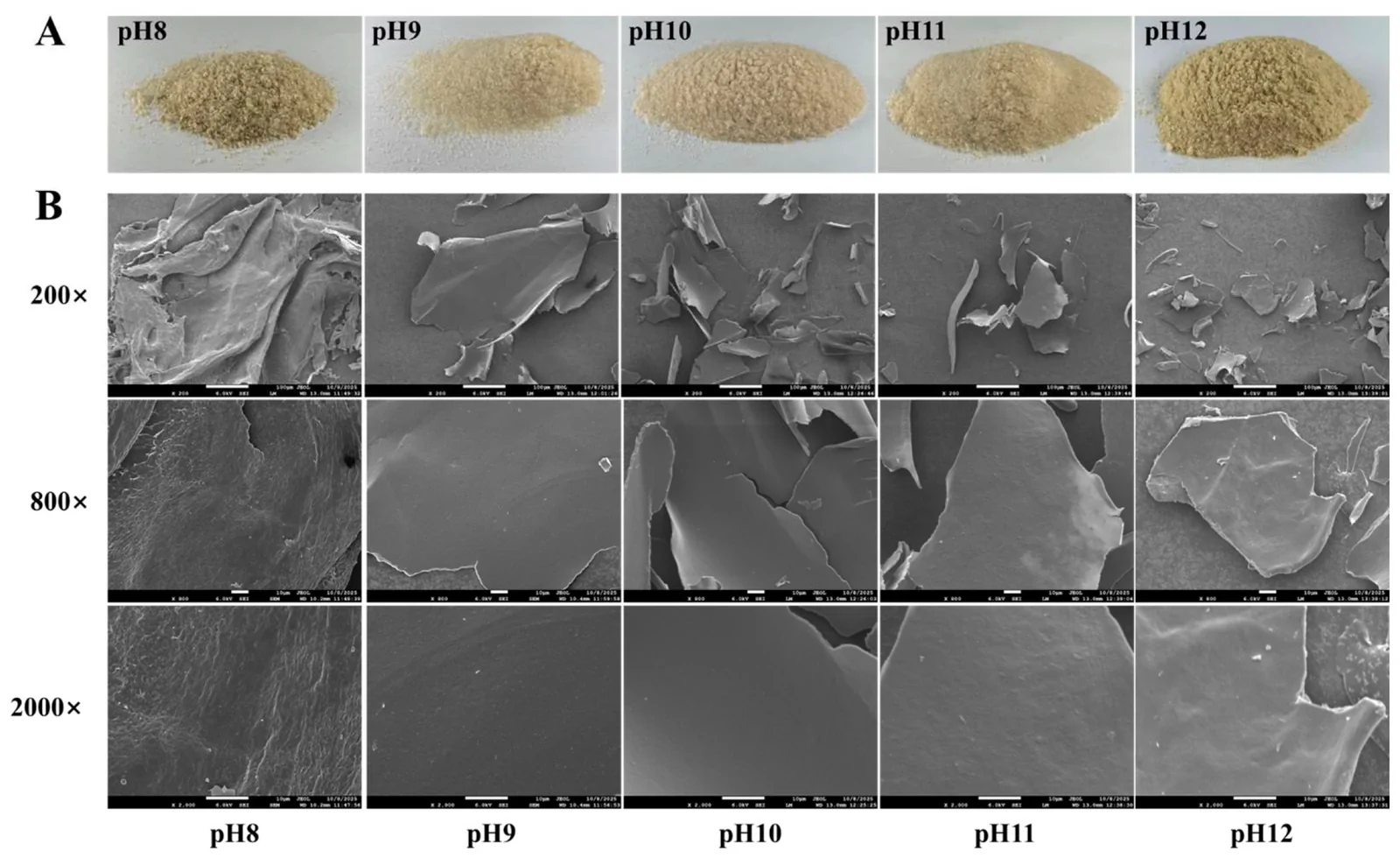
Visual appearance (**A**) and microstructure (**B**) of SMPIs with different extraction pH.

The color characteristics and visible appearance of SMPIs are presented in Table 1 and Figure 2A. Significant differences (*p* < 0.05) were observed between color parameters. As the pH increased from 8 to 12, the L* value first increased and then decreased, peaking at 89.18 at pH 10 and then to 79.69 at pH 11. Additionally, the highest a* value (4.23) was observed at pH 10. From the appearance of the SMPIs, the color of the protein exhibited a distinct deep yellow color at pH 12 isolates. From this, it appeared that extreme alkaline extraction (pH 12) increased the b* value (yellow value) to 23.54. Under certain alkaline conditions, pigment substances in the mantle are readily dissolved and co-precipitate with mantle proteins, leading to a decrease in the L* value and an increase in the b* value of SMPIs, ultimately resulting in a darkening of SMPI color [29]. The highly alkaline conditions may intensify protein denaturation (affecting light scattering) [1]. Singh et al. [30] reported that the yellow appearance of isolated proteins might be associated with lipid retention during extraction.

### 3.4. Microstructure Analysis

Figure 2B exhibits the microstructure of SMPIs under different pH conditions. At low alkaline extraction conditions (pH 8), SMPIs had a rough surface and an irregular geometric structure. Notably, proteins showed a layered morphology under high alkaline treatment, with relatively smooth and compact surfaces. This may be attributed to the unfolding of proteins induced by high pH, which increased the probability of disulfide cross-linking. Additionally, SMPIs obtained through high alkaline extraction had higher purity and presented a denser morphology after freeze-drying. Similar results were reported by Liu et al. [1], who found that oyster protein isolate extracted at higher alkalinity (pH 10~12) exhibited smoother surfaces compared to those extracted under lower alkalinity conditions (pH 8~9). Another notable feature was that the microstructure of SMPIs under higher alkaline conditions exhibited flakes of smaller sizes. Our alkali extraction method obviously differed from the microstructure (rough, wrinkled, and folded) of scallop mantle proteins extracted enzymatically [31].

### 3.5. DSC Analysis

**Figure 3:**
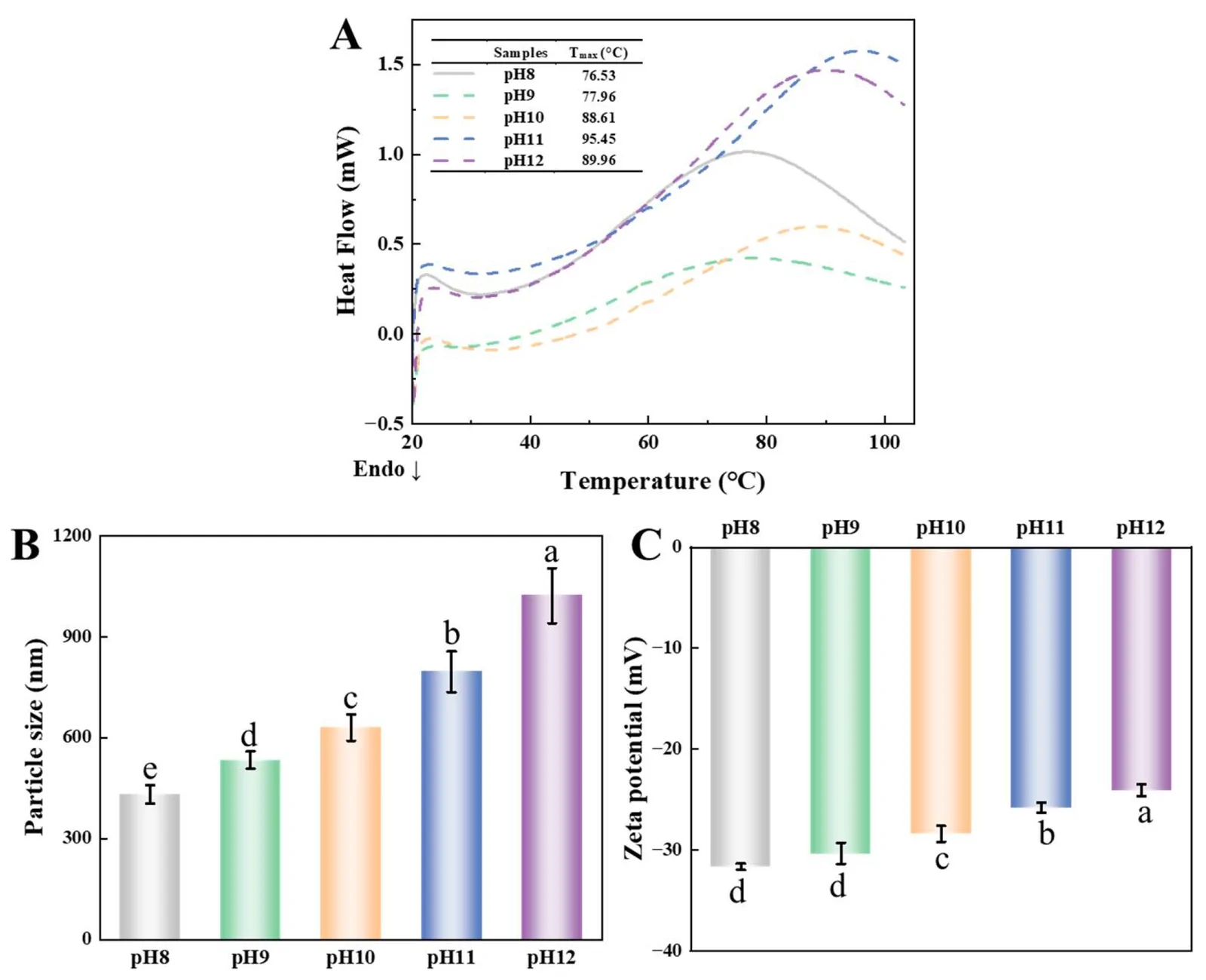
DSC (**A**), particle size (**B**), and zeta potential (**C**) of SMPIs with different extraction pH. DSC, differential scanning calorimetry. Different letters indicate significant differences (*p* < 0.05).

The improvement in protein thermal stability broadens the scope of thermal processing [32]. The thermal stability of proteins was monitored by estimating their maximum denaturation temperature (T_max_) via differential scanning calorimetry (DSC). As illustrated in Figure 3A, all samples exhibited only a distinct endothermic peak, which could be attributed to denaturation resulting from protein unfolding during heating. The increased extraction pH (from 8 to 11) elevated the maximum denaturation temperature of SMPIs (from 76.53 to 95.45 °C), but it decreased at pH 12 (89.96 °C). This was similar to the report by Liu et al. [31], who indicated that the denaturation temperature range for the scallop (*Chlamys nobilis*) mantle protein was between 70 and 80 °C. The denaturation temperature (T_max_) unexpectedly decreased from 95.45 °C at pH 11 to 89.96 °C at pH 12, which may indeed be attributable to severe chemical degradation of the protein backbone under extremely alkaline conditions (Figure 1C). Typically, proteins with high denaturation temperatures have favorable thermal stability. The increase in denaturation temperature was correlated with enhanced intermolecular interactions, which arose from the exposure of hydrophilic and hydrophobic sites within the polypeptide chain [25]. Nevertheless, protein rearrangement by extreme pH may result in excessive aggregation and occlusion of active sites, ultimately reducing intermolecular interactions and thermal stability [1]. Furthermore, elevated extraction pH may disrupt hydrogen bonds, consequently lowering its thermal stability [25]. Xu et al. [28] claimed that silkworm pupa protein isolates prepared under alkaline conditions exhibited greater thermal stability than those prepared under acidic conditions. Overall, the increased pH during extraction enhanced the thermal tolerance of SMPIs, particularly at pH 11.

### 3.6. Particle Size, PDI, and Zeta Potential Analysis

The particle size distribution of SMPIs was determined using dynamic light scattering (DLS) to investigate protein aggregation. The average particle size of SMPI samples exhibited significant variation (*p* < 0.05), ranging from 431.75 to 1021.87 nm (Figure 3B). The SMPI sample extracted at pH 8 displayed the smallest particle size (431.75 nm). A further increase in extraction pH progressively enlarged the SMPI particle size. This indicated that higher alkaline extraction conditions facilitated the aggregation of scallop mantle protein molecules into larger particles. These results were consistent with findings from Liu et al. [1] on oyster protein isolates and attributed to the exposure of hydrophobic groups within proteins during alkaline extraction treatment. The subsequent protein aggregation driven by hydrophobic interactions likely increased the particle size of proteins. PDI is a kind of symbol to represent the degree of stability, and it could characterize stability in particle size distribution. As shown in Table S1, the particle size distribution for all groups remained within acceptable limits, whilst SMPIs extracted under pH 10 conditions exhibited the highest stability. Furthermore, previous studies have shown that smaller protein aggregates exhibit higher solubility and superior functional properties due to the larger interaction area between the protein and water molecules [33].

The zeta potential can reflect the surface charge state of proteins, which serves as an indicator of the strength of electrostatic interactions between particles [34]. Figure 3C shows that all SMPI samples were negatively charged due to the extraction environment above their isoelectric point, with a noticeable decrease of the absolute zeta potential values when the extraction pH increased. This decrease could be explained by amino and carboxyl group deprotonation, thereby exposing or hiding the binding sites and affecting the zeta potential [25]. Systems with lower absolute zeta potential values exhibited weaker repulsive forces and higher propensity for aggregation [35]. Consequently, the reduction in the absolute zeta potential values of SMPIs caused by elevated alkaline extraction pH values led to increased protein aggregation, larger particle size, and diminished solubility. Here, the diminished absolute zeta potential value provided support for the findings related to particle size measurements. Notably, the zeta potential of proteins may be influenced not only by pH extraction but also protein conformation and particle size [36]. Hence, no significant variation in the absolute zeta potential values was observed between pH 8 and 9 (*p* > 0.05). The extreme pH extraction reduces net surface charge stability, enabling unfolded hydrophobic peptide chains to overcome repulsion barriers and assemble into large aggregates upon neutralization.

### 3.7. SMPI Structural Properties Analysis

#### 3.7.1. Surface Hydrophobicity

**Figure 4:**
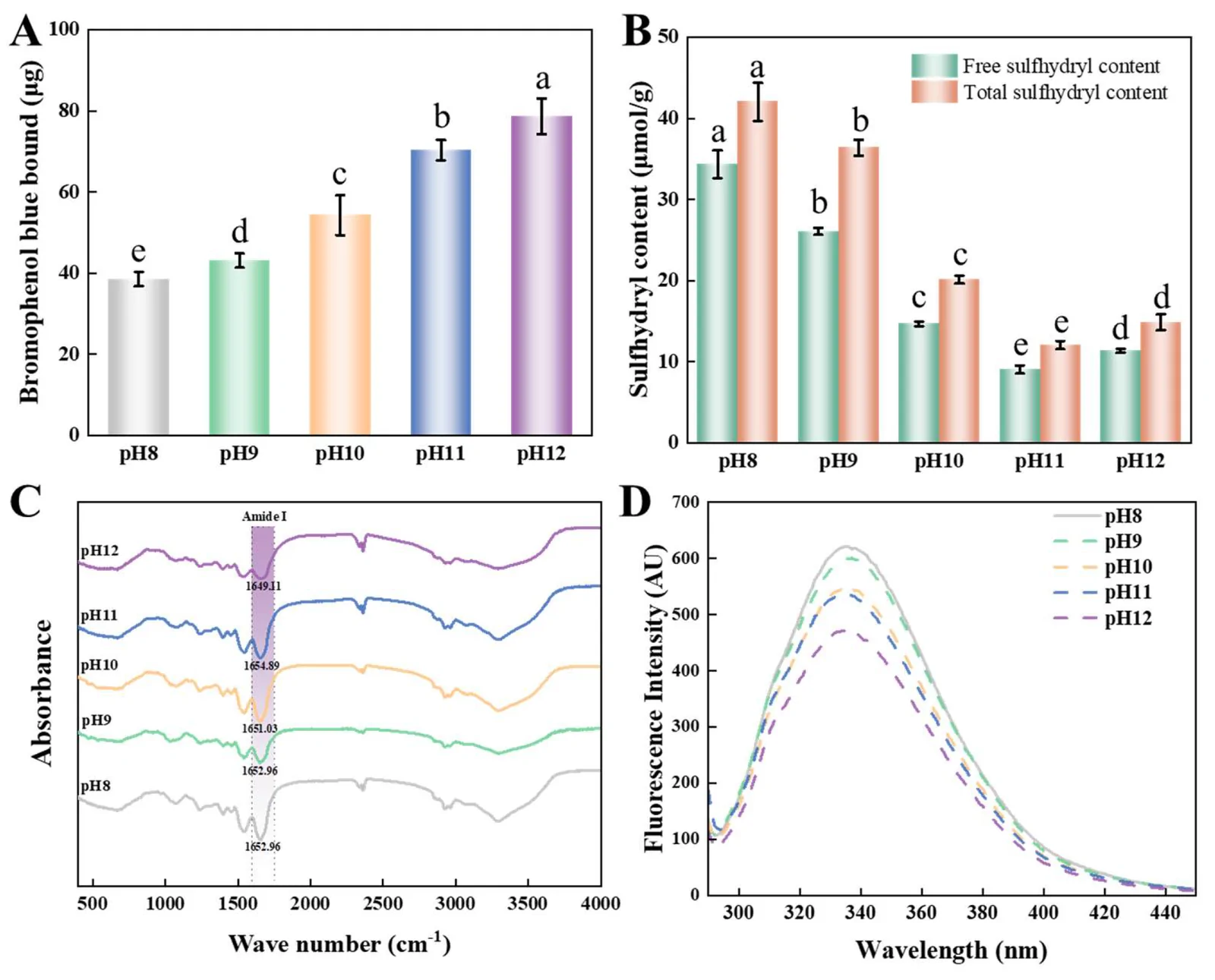
Surface hydrophobicity (**A**), sulfhydryl content (**B**), FTIR spectra (**C**), and intrinsic fluorescence spectrum (**D**) of SMPIs with different extraction pH. FTIR, Fourier transform infrared spectroscopy. Different letters indicate significant differences (*p* < 0.05).

Surface hydrophobicity acts as a measure of the exposure level of hydrophobic groups within proteins, offering insights into alterations in protein structure. As elucidated in Figure 4A, the bromophenol blue bound of SMPIs increased significantly from 38.54 μg (pH 8) to 78.63 μg (pH 12) with rising extraction pH. This indicated that protein structure unfolding induced by alkaline extraction caused more hydrophobic group exposure on the protein surface and consequently increased surface hydrophobicity [22]. It has been reported that the isoelectric points (pI) of scallop gonad protein isolates and mantle proteins are pH 3.8 and pH 4.5–5.0, respectively [37]. Generally, when the pH deviates significantly from the pI value, the electrostatic repulsion between protein molecules is intensified, predisposing them to protein unfolding [38]. Previous reports have indicated that the exposure of hydrophobic regions in proteins is directly related to reduced solubility [39], which is consistent with our solubility results. Similar results were observed by Liu et al. [1] for pH-treated oyster protein isolates, indicating that pH changes involve protein structural rearrangement and exposure of hydrophobic groups. Additional support for these findings came from the particle size and zeta potential results.

#### 3.7.2. Free and Total Sulfhydryl Contents

Changes in the sulfhydryl (SH) groups of proteins imply alterations in the internal structure of the protein molecule, thereby affecting its functionality [35]. Figure 4B illustrates the effect of the alkaline pH extraction process on the SH content of SMPIs. As the extraction pH increased, the free SH content of SMPIs decreased significantly, from 34.31 μmol/g at pH 8 to 11.35 μmol/g at pH 11. The total SH group also exhibited an identical trend of change. Our observations indicated that SMPIs’ molecular chains extracted under extreme alkaline pH conditions were more prone to unfolding (Figure 4A). Hence, the decrease in free and total SH contents may be attributed to protein unfolding during extraction, which exposed internal sulfhydryl groups within the protein and formed disulfide bonds through intermolecular or intramolecular interactions [1]. The final result was a decrease in SH content and a more compact protein structure. This reduction was consistent with the observation of oyster protein isolates [1] and guar germ proteins [25] under alkaline pH conditions. On the other hand, when the extraction pH was higher than 11, the free and total SH content of SMPIs significantly increased. This could be linked to cleavage of disulfide bond in the strong alkaline environment promoting the dissociation of the protein [35,40]. The covalent bonds were strengthened by exposed sulfhydryl groups in the polypeptide chain, ultimately leading to an elevated denaturation temperature (Figure 3A).

#### 3.7.3. FTIR Spectra

To investigate the secondary conformational changes in SMPIs after alkaline extraction, FTIR spectra under different treatment conditions are presented in Figure 4C. The amide I region (1600–1700 cm^−1^) is characterized by C=O stretching vibrations, serving as a sensitive indicator of secondary structural changes [21]. The secondary structure content of each sample was calculated through second derivative analysis and Gaussian curve fitting on the spectrum at amide I (Table 2).

We found that the α-helix content of SMPIs gradually decreased with the increase in extraction pH from 20.37% to 16.59%. Another prominent finding was a significant increase in β-sheet content. It was proposed that higher alkaline extraction conditions result in a shift from α-helix and β-turn structures to β-sheet structures of the isolated proteins [1]. Compared to pH 8, light alkaline extraction significantly elevated β-turn content, though no difference was observed from pH 9 to 12 (*p* > 0.05). Previous studies have shown that intramolecular hydrogen bonds played a crucial role in stabilizing the α-helix structure of proteins, which was essential for preserving native conformation [41]. The stability of the α-helix is primarily attributed to hydrogen bonds formed between the carbonyl oxygen (C=O) and the hydrogen of the amino group (-NH) [42]. Conversely, the stability of the β-sheet structure is associated with the hydrogen bonding interactions between adjacent peptide chains, which is a crucial conformation for protein aggregation. Therefore, the reason for this discrepancy might lie in the disruption of hydrogen bonds that maintained protein secondary structures under alkaline conditions, leading to the unfolding of α-helix structures and conversion to β-sheet. In specific conditions, unfolded protein molecules can undergo structural rearrangements to produce secondary structures with greater thermodynamic stability (Figure 3A), such as β-sheets [43]. These results were also consistent with the increased surface hydrophobicity observed in this study (Figure 4A).

#### 3.7.4. Intrinsic Fluorescence Spectroscopy

Intrinsic fluorescence spectroscopy is a technique to track the tertiary structural changes of proteins based on the fluorescent residues of tryptophan. As seen in Figure 4D, the maximum fluorescence emission wavelength (λ_max_) of SMPIs was located at 333–337 nm. Typically, tryptophan is situated in a ‘polar’ microenvironment when λ_max_ > 330 nm [11]. Therefore, the tryptophan residues in SMPIs after alkaline extraction were positioned within a ‘polar’ microenvironment. When more tryptophan residues buried within the protein core are exposed to a polar microenvironment and quenched, this manifests as a reduction in intrinsic fluorescence intensity [20]. SMPIs obtained under milder alkaline extraction conditions (pH 8 and 9) exhibited higher intrinsic fluorescence intensity. The increased pH during extraction resulted in a gradual decreased fluorescence intensity, which could be attributed to prolific unfolding of the SMPIs’ conformation induced by highly alkaline extraction. As a result, more tryptophan residues and hydrophobic groups that initially buried within the protein were exposed to a more polar environment as the extraction pH increased [34]. During extraction, the peptide chains of SMPIs underwent extension, and the proportion of α-helix decreased, consistent with the results of FTIR analysis [44]. These results demonstrated that the tertiary structure of proteins was affected by pH changes.

### 3.8. SMPI Functional Property Analysis

#### 3.8.1. Solubility

**Figure 5:**
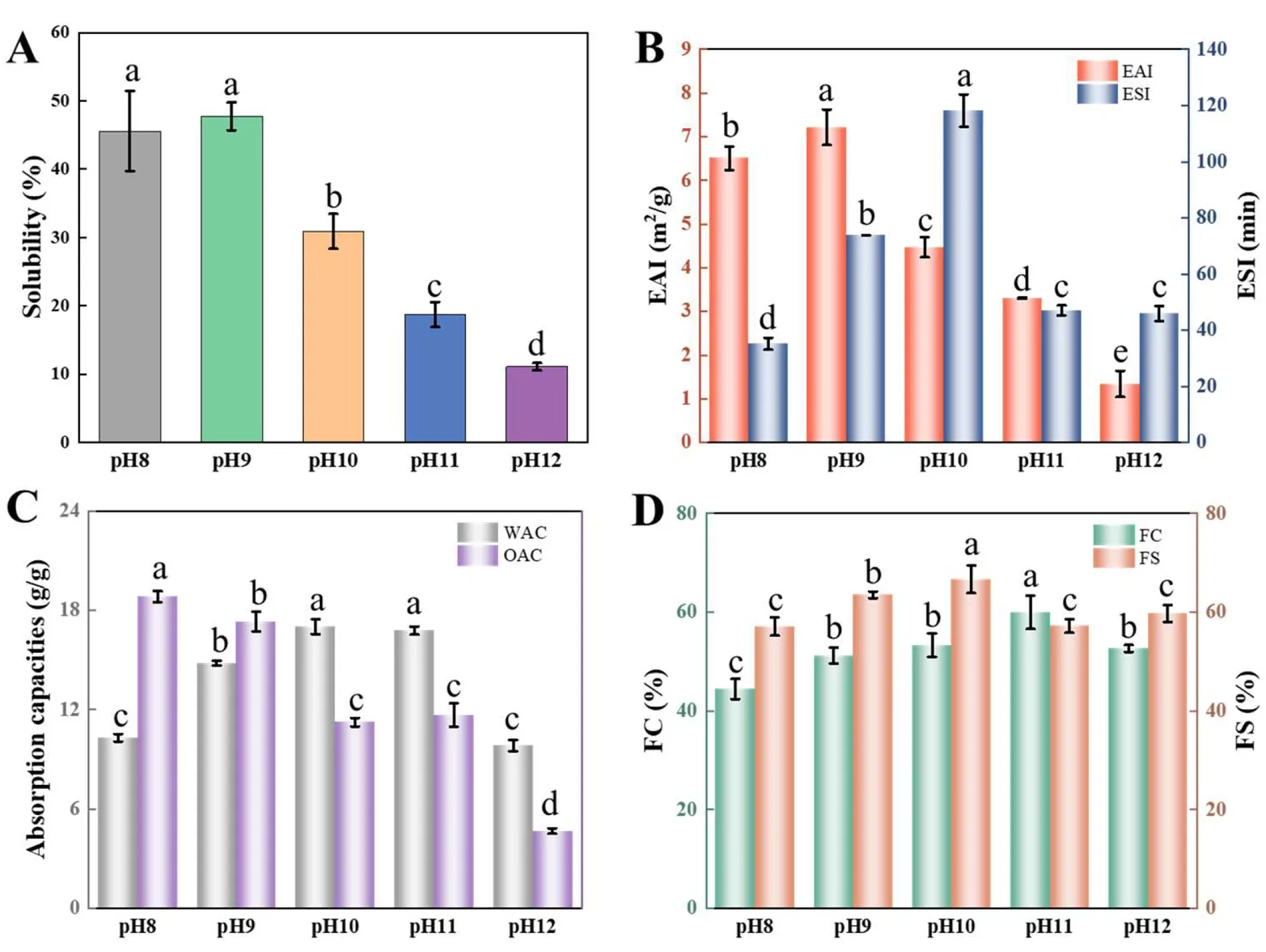
Solubility (**A**), emulsifying properties (**B**), WAC and OAC (**C**), and foaming properties (**D**) of SMPIs with different extraction pH. EAI, emulsifying activity index; ESI, emulsifying stability index; WAC, water absorption capacity; OAC, oil absorption capacity; FC, foaming capacity; FS, foaming stability. Different letters indicate significant differences (*p* < 0.05).

Solubility indirectly correlates with the protein aggregation state and can significantly influence its functional properties, such as emulsifying and foaming properties. As regards the relationship between the solubility of protein isolates and pH, a typical inverse trend was observed with a minimum solubility at pH 12, approximately 11.05 (Figure 5A). This may be explained by the fact that the higher alkaline pH promoted the protein denaturation and aggregation of SMPIs’ aggregation, with their natural state proving difficult to restore upon resuspension. During alkaline extraction, the decrease in the absolute zeta potential value of the sample (Section 3.6) led to protein aggregation and hence the observed reduction in solubility [35]. On the other hand, an increase in protein surface hydrophobicity was associated with a decrease in SMPIs’ solubility. The exposure of hydrophobic amino acids on the protein surface promoted extensive intermolecular interactions, leading to reduced solubility [39]. Notably, the addition of acid to neutralize the solution during the protein extraction process results in the creation of salts. These salts naturally push the protein out of the water, thereby increasing the likelihood of aggregation. Similarly, the solubility of the oyster protein isolate we observed earlier decreased from 53.54% to 19.91% when the extraction pH was raised from 8 to 12 [1]. It was noteworthy that protein solubility typically exhibited a U-shaped pH–solubility profile in which the lowest solubility was seen at isoelectric point (pI) [45]. This was inconsistent with the results presented in our study. Aside from protein denaturation, certain insoluble protein aggregates can be extracted under highly alkaline conditions. However, this aggregation may remain insoluble when the conditions are neutralized, resulting in lower protein solubility [46].

#### 3.8.2. Emulsifying Properties

Excellent emulsifying properties are crucial for expanding the application of protein foods like beverages, bread, condiments, and plasticizers. The emulsifying activity index (EAI) and emulsifying stability index (ESI) of SMPIs at different extraction pH are illustrated in Figure 5B. The highest EAI (7.21 m^2^/g) was noted at pH 9, representing an increase of 0.71 m^2^/g compared to pH 8. Emulsifying properties were closely related to protein solubility [23], and hence the highest protein solubility at this point also occurred. The observed increase in EAI might be due to the partial expansion and exposure of hydrophobic groups, which improved surface hydrophobicity, interfacial activity, and molecular flexibility [47], aligned with the results presented in Section 3.7.1 of this study. Similarly, the ESI of SMPIs also increased significantly, reaching a peak at pH 10 (118.16 min). This aligned with the established understanding that protein unfolding in alkaline conditions and increased peptide chain flexibility contribute to the stabilization of oil-in-water emulsions. Nevertheless, the higher alkaline conditions used during protein extraction induced protein conformation severe denaturation and aggregation, thereby negating the advantages conferred by enhanced flexibility. Gong et al. [48] noted that protein molecules with smaller particle sizes exhibited superior emulsifying properties due to their reduced size upon binding with oil droplets. Moreover, the higher net surface charge exerted a stabilizing effect on oil droplets and prevented emulsion droplet aggregation, which enabled proteins to disperse uniformly across the oil–water interface [49]. Consequently, the emulsifying capacity of SMPIs diminished under higher alkalinity conditions. Overall, appropriately alkaline extraction application (pH 9~10) effectively enhanced the emulsifying properties of the extracted protein, thus broadening its prospects for extensive application within the emulsifier market.

#### 3.8.3. WAC and OAC

As shown in Figure 5C, the WAC of SMPIs exhibited a trend of initially increasing and then decreasing with the increase in extraction pH. The maximum WAC value of SMPIs occurred at pH 10 (17.00 g/g), with no significant difference compared with pH 11 (*p* > 0.05). The partial unfolding of proteins during alkaline treatment led to greater exposure of polar amino acid groups, which exhibited heightened affinity for water molecules, ultimately resulting in enhanced WAC of SMPIs [50]. However, higher extraction pH values (>10) may diminish the water-absorbing properties due to excessive aggregation. Extreme alkaline extraction conditions (pH > 10) may diminish WAC properties due to excessive aggregation [1]. Notably, the OAC of SMPIs decreased significantly with increasing extraction pH. This indicated that alkaline extraction reduced the oil absorption capacity of SMPIs, which may compromise flavor, taste, and mouthfeel during food preparation. The inverse trend between surface hydrophobicity and OAC presents an interesting phenomenon that was resolved through physical microstructural analysis (Figure 2B). After freeze-drying, this smooth and compact surface of the protein reduced the penetration of oil molecules into the protein. This loss of porous structure severely diminished the capillary forces required to draw and physically entrap oil droplets within the powder matrix. Consequently, despite the greater exposure of hydrophobic groups, the absence of micro-capillary networks acted as the primary limiting factor, resulting in a significant reduction in OAC at higher extraction pH. At moderate alkaline extraction conditions (pH < 10), the OAC of SMPIs significantly exceeded WAC, indicating pronounced hydrophobicity. Conversely, differing outcomes were observed at extreme extraction pH values. In the future, the extraction pH value may be appropriately adjusted based on the application scenarios of SMPIs in food products.

#### 3.8.4. Foaming Properties

Foams are essential in many food formulations due to their exceptional ability to contribute distinctive textures, visual appeal, and flavors to the final products. Therefore, the foaming capacity (FC) and foaming stability (FS) of protein isolates treated with alkaline extraction were determined to assess the impact of extraction pH on the protein functional properties (Figure 5D). The FC of SMPIs extracted at pH 8 was 44.47%, while the FC of SMPIs extracted under the alkaline extraction increased first and then decreased. It reached the maximum value of 60.02% at pH 11. Furthermore, the FS of SMPIs exhibited a similar trend, with the samples at pH 10 displaying significantly higher FS values than those under other treatment conditions (*p* < 0.05). The results indicated that moderately alkaline extraction conditions could enhance the foaming properties of SMPIs. A recent study has shown that oyster proteins extracted through mild alkaline extraction (pH 10) exhibit optimal performance in terms of foaming capacity and stability [1]. Surprisingly, the solubility of SMPIs in this study gradually decreased as the extraction pH increased, yet proteins extracted at pH 10–11 still exhibited high foaming capacity and stability. Although hydrophobic exposure induced at pH 10–11 triggers self-aggregation in the bulk aqueous phase (reducing solubility), these exposed hydrophobic domains and flexible peptide chains rapidly cross-link at the interface to form a thick, viscoelastic, protective film that effectively resists film drainage and coalescence (thus improving stability) [51]. The observed decrease in FC and FS was attributed to the persistent exposure of hydrophobic groups within the protein during extreme alkaline extraction, which intensified pH-induced aggregation behavior and diminished protein interfacial activity [52]. Overall, the excellent foam stability (pH 10–11) was advantageous for the preparation of products such as ice cream and bread.

## 4. Conclusions

In this study, the effect of extraction pH (8–12) on structural and functional properties of SMPIs was investigated. It was found that extraction conditions at higher alkaline levels could significantly improve the yield, purity, particle size, and surface hydrophobicity of SMPIs. In particular, the yield of SMPIs increased significantly from 5.01% to 96.29% when the extraction pH was raised from 8.0 to 12.0, whilst purity also improved (reaching 96.01% at pH 10.0 before plateauing). Nevertheless, the absolute zeta potential, sulfhydryl content, solubility, and oil absorption capacity generally showed a descending trend. The α-helix structure was unfolded and converted into β-sheet during high pH extraction. Concurrently, internal tryptophan residues became exposed and molecular weight distribution changed, but smooth and compact microstructure was observed. Notably, SMPIs extracted at pH 11 exhibited superior thermal stability and foaming capacity (FC). SMPIs obtained at pH 10 had optimal water absorption capacity (WAC), emulsifying stability index (ESI), and foaming stability (FS). At pH 8, the SMPI exhibited minimal particle size, optimal solubility, and oil absorption capacity (OAC). In conclusion, it could be posited that pH 12 may provide a higher extraction yield, whereas pH 10–11 appears to provide a better balance between extraction efficiency and selected functional properties. Notably, the excess salts introduced during the extraction process in industrial production must be removed to ensure the standardization of functional properties and environmental sustainability.

## Figures and Tables

**Table 1:** Color values of SMPIs with different extraction pH.

Conditions	L* (Lightness)	a* (Green–Red)	b* (Blue–Yellow)
pH8	81.64 ± 1.18 ^c^	2.46 ± 0.21 ^c^	16.62 ± 0.58 ^b^
pH9	84.86 ± 1.30 ^b^	2.03 ± 0.25 ^c^	15.38 ± 0.38 ^c^
pH10	89.18 ± 1.38 ^a^	4.23 ± 0.22 ^a^	14.69 ± 0.73 ^c^
pH11	84.13 ± 1.27 ^b^	2.48 ± 0.30 ^c^	16.06 ± 0.71 ^bc^
pH12	79.69 ± 0.94 ^d^	3.74 ± 0.41 ^b^	23.54 ± 0.96 ^a^

Results are mean ± SD. Different letters in the same column indicate a statistically significant difference (*p* < 0.05).

**Table 2:** The secondary structure content (%) changes of SMPIs with different treatment conditions.

Conditions	α-Helix	β-Sheet	β-Turn	Random Coil
pH8	20.37 ± 0.25 ^a^	28.50 ± 0.18 ^e^	30.63 ± 0.16 ^c^	20.50 ± 0.35 ^a^
pH9	19.38 ± 0.46 ^b^	29.63 ± 0.18 ^d^	35.74 ± 0.47 ^b^	15.25 ± 0.74 ^d^
pH10	17.44 ± 0.03 ^c^	30.06 ± 0.13 ^c^	35.71 ± 0.12 ^b^	16.79 ± 0.05 ^b^
pH11	17.35 ± 0.05 ^c^	30.78 ± 0.02 ^b^	35.79 ± 0.04 ^b^	16.08 ± 0.04 ^c^
pH12	16.59 ± 0.67 ^d^	32.81 ± 0.35 ^a^	35.33 ± 0.35 ^b^	15.26 ± 0.18 ^d^

Results are mean ± SD. Different letters in the same column indicate a statistically significant difference (*p* < 0.05).

## Data Availability

The original contributions presented in this study are included in the article/Supplementary Material. Further inquiries can be directed to the corresponding author.

## Supplementary Materials

The following supporting information can be downloaded at https://www.mdpi.com/article/10.3390/foods15183352/s1, Table S1. PDI of SMPIs with different extraction pH.
