# Supplementary material for: Strategic Modification of Scallop Mantle Protein Isolates via Alkaline Extraction: Resource Utilization of Biological Waste
Source: Foods. 2026 Sep 21;15(18):3352. doi: 10.3390/foods15183352 (PMC13606834; doi:10.3390/foods15183352)
Supplement: Supplementary file 1 [file foods-15-03352-s001.zip › foods-4550016-supplementary.pdf]

Table S1. PDI of SMPIs with different extraction pH.

**Conditions**	pH8	pH9	pH10	pH11	pH12
PDI	0.37 ± 0.01	0.44 ± 0.03	0.23 ± 0.03	0.51 ± 0.08	0.53 ± 0.06
